# Exploring the Use of Information and Communication Technology by People With Mood Disorder: A Systematic Review and Metasynthesis

**DOI:** 10.2196/mental.5966

**Published:** 2016-07-01

**Authors:** Hamish Fulford, Linda McSwiggan, Thilo Kroll, Stephen MacGillivray

**Affiliations:** ^1^School of Nursing and Health SciencesUniversity of DundeeDundeeUnited Kingdom; ^2^Social Dimensions of Health Institute (SDHI)Universities of Dundee and St AndrewsDundeeUnited Kingdom; ^3^School of Nursing and Health SciencesCentre for Health and Related ResearchUniversity of DundeeDundeeUnited Kingdom

**Keywords:** information and communication technology, ICTs, mood disorder, metasynthesis, self-management

## Abstract

**Background:**

There is a growing body of evidence relating to how information and communication technology (ICT) can be used to support people with physical health conditions. Less is known regarding mental health, and in particular, mood disorder.

**Objective:**

To conduct a metasynthesis of all qualitative studies exploring the use of ICTs by people with mood disorder.

**Methods:**

Searches were run in eight electronic databases using a systematic search strategy. Qualitative and mixed-method studies published in English between 2007 and 2014 were included. Thematic synthesis was used to interpret and synthesis the results of the included studies.

**Results:**

Thirty-four studies were included in the synthesis. The methodological design of the studies was qualitative or mixed-methods. A global assessment of study quality identified 22 studies as strong and 12 weak with most having a typology of findings either at topical or thematic survey levels of data transformation. A typology of ICT use by people with mood disorder was created as a result of synthesis.

**Conclusions:**

The systematic review and metasynthesis clearly identified a gap in the research literature as no studies were identified, which specifically researched how people with mood disorder use mobile ICT. Further qualitative research is recommended to understand the meaning this type of technology holds for people. Such research might provide valuable information on how people use mobile technology in their lives in general and also, more specifically, how they are being used to help with their mood disorders.

## Introduction

Mood disorder is a diagnostic category containing, among others, diagnoses such as major depression and bipolar depression [1]. For some, having a mood disorder can be a lifelong problem and the need to support people with such long-term conditions is a major challenge facing health care providers. In order to effectively manage their health and wellbeing, people with a mood disorders may have to master a range of skills and make lifestyle changes, either independently or with the support of others, such as family, friends, third sector services, and mental health and social care professionals [2].

In mental health care systems designed primarily to treat acute episodes of care, the rise in long-term conditions has threatened the sustainability of services and ultimately failed to meet the needs of patients with ongoing care management and the delivery of psychosocial interventions [3]. Developments in information and communication technology (ICT) (such as the use of the Internet or computer technology and electronic systems) have begun to provide new ways for people to manage their health. ICT interventions have become affordable, accessible, and versatile such as through the use of Web-based self-help resources [4]. Psychological interventions have been effectively delivered through ICTs [5] while having the ability to reach rural areas within diverse populations and settings [6].

ICTs are increasingly being used for direct patient care [7]. eHealth is the umbrella term used for a broad range of health informatics applications that facilitate the management and delivery of health-related care, including the dissemination of health-related information, storage, and exchange of clinical data, interprofessional communication, computer-based support, patient-provider interaction, education, health service management, health communities, and telemedicine, among other functions [8].

In mental health care, eHealth technologies can facilitate the delivery of a wide range of effective treatments for a variety of clinical problems. They have widened the choices available to patients for selecting an approach best suited to manage their long-term condition [7]. Such choices include: computerized cognitive behavioral therapy (cCBT) [9]; computerized bibliotherapy, and Web-based self-help resources/patient information websites [10]; online counselling [11]; patient forums, blogs, social media/social networking sites (SNSs) [12], and online self-management groups [13].

More recently, a shift has occurred toward making technologies more portable or mobile, evidenced by the recent rise in smartphone and tablet ownership and usage [14]. Consequently, mHealth has become important for the delivery of health and health services. Improvements in reliability and broadband coverage means greater and faster Internet access for these mobile technologies. As a result, mobile devices have changed the way that consumers manage their health and engage with the health care system [15].

Evidence suggests that mHealth can facilitate the provision of effective interventions and support the self-management of long-term conditions [15]. Self-management is an interactive, dynamic, and daily set of activities by which people manage their long-term condition through overlapping skills, tasks, and processes. [16].

However, despite its growing popularity over the last decade, systematic research on the use of mHealth as a means of improving health outcomes remains scarce [17,18]. Many mHealth development studies to date, mostly outcome studies and randomized control trials (RCT), have failed to include patients or end users in a meaningful way or considered them only in limited ways in the design process [5,14,19-22]. This oversight has contributed to technology redundancy and abandonment rates [23]. Qualitative research that provides a more in-depth understanding of users’ views and experiences of how eHealth and mHealth affects their lives [23,24] is of vital importance if we are to understand how people use or benefit from technology and what drives them to engage, or not, with these technologies.

With the fast accumulation of qualitative studies in practice disciplines that specifically reflect experiences and subjective perspectives there is a need to bring together evidence from these studies [25]. We therefore conducted a systematic review in order to collect and synthesize all qualitative evidence exploring the use of ICTs and/or mobile information and communication technologies (mICTs) by people with mood disorder. We sought to answer the following review questions:

1. Why do people with mood disorders use (m)ICTs?

2. What are (m)ICTs being used for by people with mood disorders?

3. What are the perceived benefits and challenges of using (m)ICTs by people with mood disorders?

4. In what ways are (m)ICTs being used for self-management by people with mood disorders?

5. What role, if any, do (m)ICTs play in terms of social relationships for people with mood disorders?

## Methods

### Rationale

A protocol for the review was published in PROSPERO (ID=CRD42014008841). The systematic review and metasynthesis drew on methods proposed by Sandelowski and Barroso [26], Thomas and Harden [27], and Barnett-Page and Thomas [28]. Qualitative research synthesis is an approach developed to make use of this proliferation of qualitative findings driven from the growth of empirical research and evidence-based practice in the 1990s [26].

### Search Strategy

Due to potential difficulties in finding qualitative research [27], a sensitive systematic search strategy was created to maximize the likelihood of finding all relevant studies. The strategy consisted of two search strings combining thesaurus terms, free-text terms, and broad-based terms: one for ICTs/mICTs and one for qualitative methods (See Textbox 1 for an example of a search). Initially there were also terms for mood disorders, however, the pilot searches identified the inclusion of this string as being too specific limiting the aggregative capabilities of the search strategy. The systematic review would therefore categorize and catalogue all qualitative health research related to ICTs with mood disorder being the category focused upon for synthesis.

**Example search of Cumulative Index to Nursing and Allied Health.**

1. (MH “World Wide Web Applications+”) (MH “Computers, Hand-Held”) (MH “Macintosh Microcomputers”) (MH “Multimedia”) OR (MH “Social Media”) (MH “Telemedicine+”) OR (MH “Telepsychiatry”) OR (MH “Telehealth+”) (MH “Computers, Portable+”) (MH “Computer Input Devices+”)OR TI App OR TX Mobile phone*OR TX Mobile Internet OR TX Sony OR TX HTC OR TX Nokia OR TX Samsung OR TX Wireless OR TI 5G OR TI 4G OR TI 3G OR TX Touch screen OR TX Context-aware system* OR TX Cel* Phone* OR TX User-centered design OR TX Mobile app* OR TX Internet treatment OR TX Virtual realit* OR TX Internet delivered OR TX Mobile technolog* OR TX Electronic health OR TX Mobile health OR TX iPad* OR TI Apple OR TX mHealth OR TX eHealth OR TX Android OR TX Blackberry OR TX Windows mobile* OR TX Windows phone* OR TX Smartphone* OR TX iPhone* OR TI Mobile*

2. (MH “Phenomenological Research”) OR (MH “Observational Methods+”) OR (MH “Patient Attitudes”) OR (MH “Ethnographic Research”) OR (MH “Constant Comparative Method”) OR (MH “Purposive Sample”) OR (MH “Qualitative Studies+”) OR (MH “Focus Groups”) OR TX Theoretical sample OR TX Qualitative research OR TX Theoretical saturation OR TX Mixed methodolog*

3. 1 AND 2

4. Limit 3 to published 2007-2014

Searches were run in eight electronic databases: Medline, Embase, Cumulative Index to Nursing and Allied Health, the psychological literature database, Applied Social Sciences Index and Abstracts, British Nursing Index, Social Sciences Citation Index, and Cochrane Library. The results from each database were exported into Endnote X7 where duplicates were removed electronically and manually. The title and abstracts of the remaining articles were exported into a Microsoft Word document and numbered ready for screening.

Additionally, to optimize qualitative article retrieval the following methods were used: footnote searching; citation searching; journal run; area scanning; and author searching. In addition, experts and key authors were contacted to identify unpublished and ongoing studies. Due to research on the mobile aspect of ICTs being an emerging field, it was envisaged that grey literature might be a valuable source of primary data. Grey literature covers a wide range of material including: reports, government publications, fact sheets, newsletters, conference proceedings, policy documents, and protocols. We therefore searched the following sources for grey literature: The New York Academy of Medicine’s Grey Literature report and Open Grey and Grey Source Index. The Journal of Medical Internet Research and Biomedcentral Psychiatry were hand searched from 2007 to present day.

### Eligibility and Screening

One reviewer screened all of the titles and abstracts for inclusion in accordance with the following inclusion criteria: study used widely accepted qualitative methods to elicit in-depth experiences with findings appearing well supported by raw data (eg, participant quotes); study sample included people with mood disorders; study sample included the use of (m)ICTs; time period of 2007 to 2014 (2007 saw the release of the first ‘smartphone’, ie, Apple’s iPhone); and English language.

To optimize the validity of the search a systematic sampling strategy was adopted, whereby 10% of results were coscreened (HF & SM/LM) to facilitate consistency of approach [29]. All questionable citations from the full search results were discussed in order to reach consensus on disposal. Full texts were retrieved for those publications that were deemed to meet inclusion criteria and those that could not be adequately assessed for inclusion with the information provided in the abstract. Two authors independently assessed the full texts for inclusion and then met to discuss their decisions. Where they could not come to a consensus, a third author was consulted.

### Quality Appraisal

There is a lack of agreement about the approach to quality appraisal in qualitative research [26,30]. Therefore, due to the scarcity of data on the topic being studied, papers were not excluded based on quality, instead all papers were included and their quality appraised. A global assessment of study quality was undertaken assessing studies as being either strong or weak. Strong studies would likely include elements of respondent validation, triangulation of data, transparency, reflexivity, clear descriptions of methodology, methods of data collection, analysis, and an overall fit in regards to the research questions and the design of the project [31]. Reports were both individually and comparatively appraised. A typology designed by Sandelowski and Barroso [32] for classifying findings was used. Rather than comparing differences in quality between studies the typology was used to identify the level of data transformation.

### Synthesis

The synthesis stage used thematic synthesis, an approach that combines elements of meta-ethnography and grounded theory providing the opportunity to synthesis methodologically heterogeneous studies [27,28]. The thematic synthesis followed a three-step process described in Textbox 2.

**Thematic synthesis steps.**

Step 1

Free sentence-by-sentence coding: the verbatim findings of each selected study were entered into NVivo 10. Codes were developed initially free from hierarchical structure but as the translation of concepts developed from one study to another new codes were either added to existing ones or new codes created.

Step2

Organization of free codes in hierarchical order under a range of descriptive themes: free codes were organized into related areas to create descriptive themes; then similarities and differences between codes were studied, facilitating the organization of the codes into related groups and the formation of a hierarchical tree structure of descriptive themes.

Step 3

Development of analytical themes: descriptive themes were analyzed and then organized into more abstract analytical themes, producing a synthesis that went beyond the data in the original studies and addressed the research questions.

In order to keep the synthesis as close to the data as possible the research questions were initially set to one side facilitating an inductive process. Codes were applied as part of an iterative process with constant comparison with other codes (Step 1). This process was repeated for all the codes until higher order categories were constructed and all codes accounted for (Step 2). The review questions were then brought to the fore and used as a framework to guide the analytical process, which focused and transformed the descriptive themes into the final synthesis (Step 3). The categorization process was examined by the reviewing team where, through discussion, changes, and adaptions were made where necessary until consensus was reached and no further changes were required. The reviewing team scrutinized the synthesis at an analytical level through a cyclical process until a final synthesis was achieved.

## Results

### Search findings

**Figure 1:**
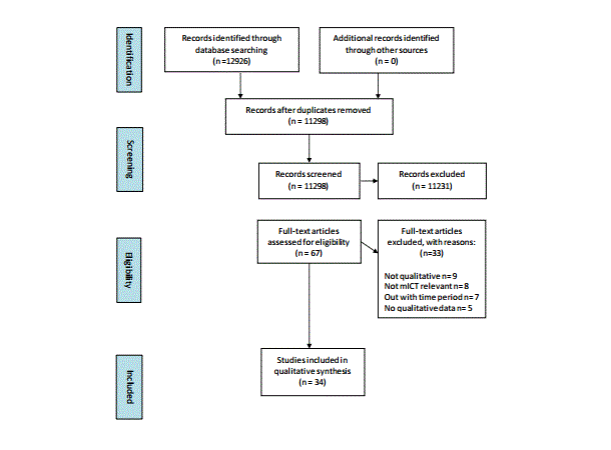
PRISMA diagram of screened articles of relevance to mood disorders and mICT .

The search identified 12,926 titles; 67 publications were retrieved in full (Figure 1). The methodological designs of the studies were qualitative or mixed-methods using focus groups, interviews, or forum/message boards as the methods for generating data. Studies originated mainly from Europe, the United States, or Australia and New Zealand.

Only one paper was identified from the systematic review of qualitative papers and therefore, synthesis of mICTs and mood disorders was not possible due to lack of data. However, the review mapped and categorized all qualitative papers in the domain of health and ICT research. This facilitated methodological development in order to find a solution regarding how to use imperfect data. Rather than lose the potentially valuable qualitative data of relevance to the project, the 67 full-text papers were rescreened. The aggregative and sensitive systematic search strategy offered a flexible approach toward the data. This provided the researchers with the ability to use the existing data to explore how people with mood disorders used ICTs ‘of relevance’ to mobile technology. This would include, but not be limited to, ICTs such as websites, online therapy, online support groups, forums, blogs, and so on, essentially, ICTs that could be accessed from mICTs but were not necessarily made explicit within the text. Thirty-four studies were included in the synthesis after the full-text articles had been rescreened; a summary of their results can be found in Multimedia Appendix 1.

The results of the appraisal process are shown in Table 1 with 22 studies identified as being strong and 12 weak, with most having a typology of findings either at the topical or thematic survey levels of data transformation. Therefore, with over a third of the studies being weak in quality, the appraised strength of the studies weighed upon, in a measured manner, our interpretation of the study findings.

The synthesis created three analytical themes and a number of respective analytical subthemes to describe people’s use of ICTs. This is presented as a typology of findings (Textbox 3).

The research questions were then used as a template, explicating the typology of findings to understand how the descriptive themes interrelated with their analytical themes, thus helping to answer the questions asked of the data. The results are presented below.

**Table 1:** Appraisal of qualitative papers in metasynthesis.

Global assessment of study quality		n (%)
	Weak	12 (35)
	Strong	22 (65)
**Typology of findings**			
	No findings	4 (12)
	Topical survey	11 (32)
	Thematic survey	14 (41)
	Conceptual thematic description	5 (15)
	Interpretive explanation	0 (0)
**Total**		34 (100)

**Typology of findings.**

Movement and change

- Change processes
- Engagement
- Motivational aspects of use
- Recovery
- Taking action
- Values

Providing a source of community

- Communication
- Intrapersonal effects
- Safe places
- Sharing
- Social aspects

The person and technology

- Acceptance of technology
- Design features
- Functionality
- Personal time
- Safety
- Technical mastery
- Technical issues
- Usability

### Why do People With Mood Disorders use (Mobile) Information and Communication Technologies?

Considerable overlap was found in terms of why people used ICTs and the perceived benefits this technology gave them. Two studies [33,34] provided data regarding motivation to use ICTs. For those involved in Internet-based treatment, the interactive nature of ICTs appeared to increase their motivation to engage with treatment and, in so doing, possibly help in their recovery [34].

Three studies showed how users of ICTs liked the option of being able to choose where to use technology (ie, at work or in the convenience of their own homes) [35-37]. Having easy access to information from around the world, at any time in the day, through use of the Internet was regarded as useful in comparison to using books [37,38].

The use of websites to support relatives of people with depression appeared to decrease feelings of stigma in both by enabling people to draw strength from talking more openly about their situation [37]. Young people were concerned about feelings of embarrassment if other people realized they had depression increasing a sense of helplessness [38-40]. Fear of being judged by others due to having a mental health issue was a particular problem faced by some young users of ICTs and it became a specific reason for using the technology [39,41]. Fear of school peers finding out and potential links to bullying opted users to engage with ICTs in the privacy of their own homes [41]. People who felt shame due to experiencing emotional problems would put a lot of effort into hiding their symptoms so having a website where people could discuss their emotional problems anonymously was considered a good thing and often would be the first time sharing their experiences [40]. The use of websites to support relatives of people with depression appeared to decrease feelings of stigma and support their mental health [41]. Using Web-based assessment tools appeared to facilitate dialogue between patients and clinicians. For instance, patients felt it was easier to talk to their general practitioner as they had thought about things beforehand and would be more confident in receiving a diagnosis [36]. Of significant importance to using ICTs was the concept of privacy [37,42-44]. Web-based programs were considered, by some, as providing the most private way to seek help for psychological issues [35].

ICTs appeared to provide people with options regarding how they used technology with choice over temporal, location, treatment, privacy, and disclosure aspects of their care needs [33,38,39,41,45]. The credibility of ICTs appeared an important factor when deciding upon usage [35]. For example, having testimonials from other users displayed on Web pages regarding credibility, and knowledge that the ICT was designed on research appeared to raise confidence in technology [35,46]. ICTs were also regarded as cost-effective solutions to access-to-care problems faced by people living in remote localities, by both patients and health professionals [47]. Participants reported being aware of long waiting times for specialist health services and, with the alternative being expensive private treatment, the low cost nature of ICTs made them attractive [34,37,42]. Modifying aspects of ICTs with a user-centered design appeared to facilitate use to some extent by reducing technical challenges and helped people feel more competent and autonomous [48,49].

### What are (Mobile) Information and Communication Technologies Being Used for by People With Mood Disorders?

The use and view of ICTs as a resource appeared to be an important factor in people’s lives. ICTs could open up access to information, support, and treatment in a highly accessible, interactive, and instant way [37,38,42,50,51]. The Internet was also considered empowering [45] and provided a resource for learning [46,48,52]. Holding certain values appeared to suggest people were going to commit to using and completing therapeutic work via ICTs more than others, for example, having a sense of what one should, or should not do, appeared to influence some people’s commitment to complete Web-based programs regardless of how tedious or frustrating they were [53]. ICTs appeared to be used as a resource for people to communicate and exchange information and stories with others [38,43,45]. They appeared to facilitate disclosure of personal information regarding people’s mental health [40], indicating a need by people to talk about their issues [34].

### What are the Perceived Benefits and Challenges of Using (Mobile) Information and Communication Technologies by People With Mood Disorders?

There was significant overlap in terms of why people use ICTs and the benefits provided. As these benefits have already been identified and discussed in the previous two sections, this section focuses on the challenges of using technology. Certain forms of technology and their functionalities produced usage difficulties [42]. That is, people were put off using ICTs if software was slow, had broken hyperlinks, was unnecessarily complex or impersonal, and if use required additional software [47,50,54]. For some people, there were concerns regarding the safety of using ICTs for treatment purposes due to queries regarding the levels of confidentiality the technology could provide [47,52,55]. Being able to use ICTs anonymously appeared to be an important aspect in managing confidentiality and a factor when assessing the appropriateness of using a Web-based intervention [37,40,43]. Indeed, there were people who preferred not to share personal information unless it was face-to-face due to the importance they placed on confidentiality [38].

Some people made a conscious decision not to use ICTs. Reasons included having no interest in certain forms of technology, not being technologically savvy, and being too unwell to use technology (for example, reduced energy and motivation due to an acute depressive phase) [38,41,56]. There were also practical reasons for not using ICTs, such as, having no need to use it, not identifying with content, inhibitive cost, or having no Internet connection [42,48]. Users needed to believe and trust in the ICTs they were using [48,52]. There were concerns regarding information reliability and quality on the Internet, and doubts as to whether people had the ability to discriminate trustworthy information themselves [45]. Of note, was the generally limited mention of negative outcomes in the reviewed studies.

### In What Ways are (Mobile) Information and Communication Technologies Being Used for Self-Management Purposes by People With Mood Disorders?

The use of ICTs appeared to support people to acquire relevant knowledge in regards to their mood problems providing a sense of recognition in situations that might be difficult to accept or unfamiliar, thus helping them feel supported [34]. ICTs were used, by some, to acquire information about treatment, diseases, drug information, and the experiences of others [44,45]. Some people read information specifically targeted toward health professionals as they deemed it to be the most comprehensive and up-to-date sources of information [45]. Seeing relevance in material appeared to be a factor in the process of acquiring new self-knowledge. This was achieved through learning together by reflecting and restructuring new knowledge to suit one’s own needs [34]. ICTs appeared to be being used for help-seeking through the acquirement of self-help information, the development of skills, and also as a means to seek help through online support groups and forums [38,40,45,57,58]. Sitting down at a computer at regular times working on a self-help programs appeared to be of benefit; people reported experiencing an empowering effect, a change of perspective, increased personal agency, and a way of keeping new learning at the forefront of their minds [46]. A programs to help monitor depression (on a mobile phone) appeared to hold potential as a motivational tool to support people to look after themselves [42]. Self-help books were viewed as having a number of disadvantages such as being hard to read, noninteractive, and difficult to engage with; where available, people preferred Web-based versions [50,59]. ICTs appeared to provide people with informational support and the ability to delve as deeply as they wished into certain topics, such as medication management, counselling services, negative thinking, and poor concentration [40,52]. The Internet appeared to be considered a key component in providing greater access to health information by patients and receiving benefits from engaging in self-help [34,40,45,46,52].

ICTs appeared to help provide a sense of control in people’s lives by providing them with the opportunity to find information about where to find help, assisted them with understanding when to seek help, and what support was available to them [38,52]. They helped prepare for meetings with health professionals, thus making treatment more collaborative [45]. Support from online forums was flexible and inexpensive [60]. Maintaining contact with friends and family was also feasible through diverse Web-based platforms [39].

Receiving support through ICTs appeared to be of benefit by people with mood disorders [49]. People required support in maintaining relationships and dealing with broken relationships while recovering from a mood disorder [50]. Methods of support, in preference to telephone calls or home visits by professionals, included the use of emails due to their unintrusive nature [49,50]. People also appeared to benefit from support from friends, family members, and significant others to encourage and help them persist with using ICTs for their recovery [48]. Web-based programs potentially offered a means to lessen stigma toward mental health and encourage acceptance of conditions such as Bipolar Disorder [35,37,52,61]. Web-based programs supported people to feel validated and empowered increasing feelings of confidence and self-worth [36,46,57].

Learning time management techniques facilitated people to organize their time better helping them meet deadlines and prepare for exams [35,48]. Time was required to be set aside to use ICTs and having personal time in a private space was appreciated [37]. People taking responsibility for their treatment, had a sense of determination, curiosity, and attributed success to their own endeavors appeared to benefit more from treatment delivered through ICTs [53,62]. People were able to use ICTs to contact health professionals and source health information for themselves, which appeared to help them manage their own problems moving from a position of passivity to one of activity [34,38,53,62]. Being aware of one’s motivational levels and having responsibility for maintaining motivation to use interventions delivered through ICTs appeared helpful [33]. Holding certain values would help people to complete Web-based programs and others found their own way of working with material to face and overcome challenges and seeing difficulties as potentially valuable lessons to be learned [53,62].

People’s awareness sometimes appeared to change when using ICTs. For instance, becoming aware of holding high expectations toward technology and feeling disappointment if programs did not meet all their needs fostered a sense of consideration to revisit and work with material to see if it would be of benefit [62,63]. Web-based programs held the potential to help people become more aware of their negative thinking habits, promoting reflection, and challenging of thoughts moving people in a more positive direction toward self-acceptance [34,37,50,64]. Participant feedback in the design process of ICTs potentially influenced the goals of particular programs centering on raising awareness regarding the importance of managing depressive symptoms among their users [65]. The use of ICTs to access information and seek other people’s opinions through online forums for example offered different viewpoints and helped people understand more about the difficulties they faced [37].

### What Role, if any, do (Mobile) Information and Communication Technologies Play in Terms of Social Relationships for People With Mood Disorders?

Using ICTs for social support appeared to be of benefit to people and one of the predominant reasons for using the technology on a daily basis [42]. Meeting similar people in virtual environments created a sense of acknowledgement and recognition decreasing feelings of social isolation, loneliness, and alienation [37,48,52]. People could use ICTs as an opportunity for sharing their feelings, emotions, and personal stories [40]. The exchange of experiences and knowledge in a supportive environment appeared to help people; narrate their experiences, gain a sense of community, share tips, and provide a sense of comfort, strength, and hope in people [37,42-45]. Using ICTs made some users less inhibited, in terms of the personal information they shared, because they felt more secure about privacy being maintained and, therefore, found ICTs less discomforting than face-to-face interactions [37,38,40].

ICTs provided people with the capacity to use online social networks in order to communicate with people experiencing similar issues, to ask advice or discuss certain topics in a convenient and accessible manner [37,38,42]. ICTs also provided people with the opportunity to receive and give peer support [37,44,50,66]. Peer support appeared to help people engage with Web-based interventions, overcome procrastination and motivational issues, and helped them to understand their own problems in a way that gave potential for behavior change [44,50].

## Discussion

### Principal Findings

The aim of the study was to conduct a metasynthesis of all qualitative studies exploring the use of ICTs by people with mood disorder. The resultant metasynthesis created an analytical typology of findings and a descriptively themed framework, which conceptualized how people with mood disorder use and relate to their ICTs, and in so doing, answered the specific review questions. The metasynthesis identified that people with mood disorders use ICTs in similar ways and face similar technological paradoxes as other users [67]. However, the metasynthesis developed the understanding further, suggesting a continuum of use, in this instance, by people with mood disorder. How ICTs of relevance to mobile technology are used and harnessed by this particular client group are discussed below.

Our metasynthesis identified the factors influencing why people with mood disorders chose to use ICTs such as affordability, accessibility, and versatility. These factors align closely with previous studies on the delivery of health-related products evidenced through increasing Web-based self-help resources [4], effective delivery of psychological interventions [68], and their ability to reach rural areas within diverse populations and settings [6]. The body of eHealth research is expanding with studies across different patient groups, using different technologies/interventions, and focusing on different outcomes [69-73]. In their interpretive review of the literature on consumer eHealth, Hordern et al [74] identified five broad usage themes: (1) peer-to-peer online support groups and health-related virtual communities, (2) self-management/self-monitoring applications, (3) decision aids, (4) the personal health record, and (5) Internet use. The results of our metasynthesis reflected these uses but also highlight a number of intrinsic factors affecting people’s use of ICTs. People were motivated to use ICTs to aid recovery, associated with the convenience afforded to them through using the technology. The privacy and choice of communication methods of ICTs were seen as facilitative and credible options often wrapped-up in cost-effective and user-center designed products. Seen as a resource, ICT use was empowering, facilitating people to self-care or self-manage. The Internet was considered a key component in providing greater access to health information by patients and for them to receive benefits from engaging in self-help [34,40,45,46,52]. ICTs helped provide a sense of control in people’s lives by providing them with the opportunity to access information to help themselves, bettered their understanding about when to seek help, and increased awareness of what help was available [38,52]. They facilitated people to prepare for meetings with their health professionals making treatment more collaborative. Receiving support through ICTs was seen to be of benefit by people with mood disorders [49]. ICTs potentially facilitated people to take the first step in managing their recovery after years of deliberation [34]. ICTs supported people to stay in contact with health professionals involved in their care and establish therapeutic relationships despite being separated by distance [38,47,49]. People were able to use ICTs to contact health professionals and source health information for themselves in order to manage their problems by converting intentions into actions [34,38,53,62].

People who took responsibility for their treatment, had a sense of determination, curiosity, and attributed success to their own endeavors appeared to benefit more from treatment delivered through ICTs [53,62]. ICTs that were stimulating to use and interacted well with peoples' senses and cognitive abilities enhanced engagement [46]; this was deemed vital in the context of online depression therapy [34]. With engagement came an enhanced sense of personal agency from interacting with ICTs and the completion of Web-based activities offered a sense of empowerment [37,46]. The functionality of ICTs was an important factor in their adoption and use [42]. For example, having functions to compare information from day-to-day to week-to-week, track information, format content, use Web-based diaries, forums, bookmarks, blogs, messages, control privacy settings, invitation functions, e-reminders, and options to reply directly to clinicians were important usage features [37,42-44,49,50]. ICTs with increased usability were desirable [38,42]. For example, having a user-interface that could be personally controlled, with an array of images, colors, information, and music options, could help engage the user [46]. In addition, being able to use ICTs in different locations (such as, work, home, or on public transport) appeared important factors when assessing usability by the user [37,42].

When designing ICTs and Web-based interventions importance was placed upon managing depressive symptoms in order to support people, through evidence-based interventions, with their practical and interpersonal issues caused by their conditions. [50,65]. Web-based programs supported users to work on solving problems through taking a structured approach using manageable steps [46,57]. If people focused on achieving manageable goals then it provided them with a sense of completion [50,53]. Web-based programs supported people to cognitively restructure, facilitating them to rethink stressful situations that challenged their negative thought patterns. Web-based programs also facilitated behavioral changes by breaking negative cycles of inactivity, self-incrimination, and withdrawal, which lead to secondary benefits as people become closer to those around them [34,35]. Peer support accessed through ICTs helped people to feel more positive and understand themselves better leading to behavior change and the confidence to negotiate changes in treatment [44]. Accessing Web-based medication information through ICT use prompted some people to request additional drug information from their prescriber regarding risks and benefits of antidepressants and conversely, made others change the dose of drug or discontinue the prescription without seeking professional guidance first [45]. Having a sense of curiosity toward ICTs and a will to learn self-management techniques and, if an improvement in their health was noticed through using ICTs, then they were more likely to persist with an intervention [48,53]. ICTs supported people to stay in contact with health professionals involved in their care and to establish therapeutic relationships despite being separated by distance [38,47,49]. However, ICTs can be viewed to have paradoxical elements to them; social and economic paradoxes, which challenged people in their social and individual lives [67].

The findings of our metasynthesis indicate that usage difficulties were a key factor in reducing people’s motivation to use ICTs. This aligns well with the findings of others, including Bessel et al [75] who identified that computer-based interventions have limitations, such as the reliance on users having access to computers at scheduled times and restricted and unreliable Internet access in remote and rural areas. Safety was a key concern raised from the synthesis with the concept of confidentiality and data security being paramount. This is clearly associated with the disadvantages of ICTs such as software errors, unreliable information, problems with privacy and unreliability, lack of regulation, social isolation, and in some forms of technology, the loss of vocal intonation and nonverbal communication [75]. Internet-delivered treatment programs such as open access websites are characterized by poor adherence with an average dropout rate of 31% [76,77].

There are many advantages for patients when using ICTs, such as being able to get in contact with health professionals quickly and easily, a reduction in travel and waiting times for face-to-face appointments, convenience, and affordability. The technology provides a medium for communication between health professionals and patients where information about the patients’ disease, treatment, and therapeutic interventions can be discussed [7]. This is of particular importance for those with long-term conditions and our metasynthesis reinforces this point. In contrast to other forms of patient contact, ICTs provide the opportunity for asynchronous communication. eHealth holds the potential to enable patients to better manage their long-term health conditions through the use of technology [7].

Our metasynthesis identified that people used ICTs to acquire relevant knowledge in regards to their mental health issues. This can be linked to an increasing trend in society to adopt self-service models of interaction. There have been promising results for using computers to deliver self-management programs to patients with long-term conditions in health-supported settings showing potential for changing health behavior and improving clinical outcomes [78]. Since 2005, interest in the Internet as a vehicle to disseminate interventions designed to treat and prevent mental disorders, including those targeted at depression, has been increasing [11]. Passive psychoeducational information might be an effective intervention for depression when employed with reminders and involving minimal information [11]. In their systematic review, Griffiths et al [11] identified that the Internet was highly effective and facilitative when used to deliver mental health interventions with or without practitioner guidance. Web-based CBT has also been shown to provide small benefits when used to help manage chronic pain [79]. However, in a recent meta-analysis of computerized cognitive behavioral therapy (cCBT) by So et al [9] only short-term reductions in symptoms were noted, long-term follow-up and functional improvement were not significant and there was a recommendation that the clinical usefulness of cCBT should be reconsidered downward in terms of methodological validity and practical implementation [9]. Therefore, further research is required to help understand peoples’ self-service approach to accessing Web-based health information and their acceptability and use of Web-based therapeutic interventions [80].

Outcome data from RCTs and meta-analyses have identified the cost-effectiveness and clinical efficacy of mental health programs delivered via ICTs with comparable effect sizes to face-to-face treatment [14,81]. Web-based interventions have also highlighted positive effects on patient empowerment and self-efficacy including people with physical health conditions such as cancer [82,83]. Our metasynthesis suggests similar outcomes for people with mood disorder and that ICTs can provide opportunities for help seeking and support for such people. Yet, evidence points to the variable quality of information and apps available on the Internet [7,84-87]. There appear to be numerous health-related websites with limited availability of help, containing information that can be difficult to read and incomplete [85,86].

Our metasynthesis identified that people with mood disorder were using ICTs to give and receive social support. This corresponds with evidence from SNS use and associated indices of psychological well-being relating to a persons’ sense of self-worth, self-esteem, satisfaction with life, and other psychological development measures [88]. The metasynthesis identified that people with mood disorder where using social networks in a similar way as suggested by Cohen [89] by providing platforms to give and receive social support in the form of informational support, instrumental support, and emotional support. The metasynthesis highlighted how ICTs facilitate people with mood disorder to communicate with others, corresponding to previous work undertaken regarding Web-based disinhibition. Suler [90] suggests that a number of factors can loosen psychological barriers allowing for inhibition to become reduced when on the Internet: invisibility, dissociative anonymity, synchronicity, dissociative imagination, solipsistic introjections, and minimizing authority. People use social networking sites (SNSs) for their sociability function to maintain relationships with on and offline friends over varying distances [91] attending to extended social networks and relationships [92,93]. Before the development of popular Web-based SNSs such as Facebook, Skinner and Zack [94] had already identified that barriers to communication were being overcome through using the Internet and as a result people were getting help in ways convenient to them.

Of particular importance was the lack of qualitative research being undertaken in this field as evidenced by only one paper retrieved specifically reporting on mICTs. To date, patients or end uses have not been sufficiently included in the design of software applications. The same applies to the selection of relevant and appropriate outcome measures in effectiveness studies such as RCTs. These omissions have contributed to redundancy and the abandonment of technology. n fact, there has been a presumption that those designing technology and undertaking research already know what the user wants in terms of software and hardware. Designers have, jumped ahead, and designed apps and websites, without first talking to end users about how they use and fit technology into both their existing lives and what would help them manage their lives. Qualitative research, which provides a more in-depth understanding of users’ views and experiences is of vital importance if we are to understand how people use or benefit from technology and what drives them to engage, or not, with these technologies.

### Recommendations

Research: research relating to how people with mood disorder used ICTs was lacking and in particular, their use of mICTs, not as participants in research studies, but as ubiquitous technology in their everyday lives. Qualitative research is required to help understand how mICTs fit into people’s lives both in general but also more specifically in relation to their mood disorders.

Practice: clinical practice could be supported through understanding how people with mood disorder use mICTs to look after themselves providing clinicians with valuable information to help harness peoples’ mICTs for use in their recovery and inform the future design of technology.

### Strengths and Limitations

The review relied exclusively upon English language publications, which may not adequately reflect the user experiences and perceptions that were gathered in non-English speaking contexts. Another issue may relate to the quality of primary data sources and the quality of existing quality appraisal tools for metasyntheses. The researcher’s stance was clearly set out in the study providing rationales for the choice of methodology and methods used. Transparency was achieved by clearly detailing the synthesis process and checks and balances were used to ensure rigor throughout. The study originally set out to synthesis all qualitative articles regarding people with mood disorder and mICT. Unfortunately, as only one article met the original inclusion criteria for mobile technology a synthesis of this material was not possible. However, our novel approach toward the search and retrieval of data allowed us to catalogue all qualitative data related to health and ICTs including data of relevance to mICTs. This process provided us with the opportunity to restructure our inclusion criteria and make use of the data that would have otherwise been neglected in other systematic reviews and metasyntheses.

### Conclusion

The metasynthesis of people with mood disorders and their use of ICTs has provided a tentative understanding into their uses, challenges, and gratifications spanning the intrapersonal, interpersonal, and through into wider society. The typology of findings and analytical framework highlights the connections and interrelationships between analytical themes and subcategories; the intrinsic and extrinsic nature of use and the embedded characteristics of the technology. Our metasynthesis has identified that people can use ICTs in novel ways to help them manage their lives and health. People use ICTs to support motivation, for their convenience, to help decrease feelings of stigma, their facilitative capabilities, enhance privacy, credibility, and cost effectiveness. ICTs support people to access the Internet to get what they need in a way that fits into their lives. ICTs are a resource for communication and promote user engagement. However, they are not without issue, with particular challenges of trust and confidentially requiring to be negotiated. That being said, when the challenges are navigated successfully, people are able access opportunities to manage their mood disorder by acquiring relevant knowledge, engage in help-seeking behavior, receive support, gain a sense of control, learn time management techniques, take responsibility, and increase their awareness. ICTs also allow access to Web-based social networks where sharing with others can facilitate social support. Our typology of findings creates an empirical basis to help guide and harness the potential of (m)ICTs to support self-management, facilitate collaborative, person-centered care, and support the person actively recover from their mood disorder. Importantly, our metasynthesis has highlighted a gap in the evidence base, as no research has focused specifically on mICT use by people with mood disorder.

## Multimedia Appendix 1

Summary of results

## Abbreviations

cCBT: computerized cognitive behavioral therapy
BD: bipolar disorder
GP: general practitioner
mICTs: mobile information and communication technologies
ICTs: information and communication technologies
RCT: randomized control trials
SNS: social networking site
