# Supplementary material for: Exploring the Use of Information and Communication Technology by People With Mood Disorder: A Systematic Review and Metasynthesis
Source: JMIR Ment Health. 2016 Jul 1;3(3):e30. doi: 10.2196/mental.5966 (PMC4947190; doi:10.2196/mental.5966)
Supplement: Supplementary file 1 [file mental_v3i3e30_app1.pdf]

Study	Method	Participants	Setting	Summary of findings	Notes
Bae J, et al [65]	Interviews and focus groups	Interviews: 14 patients.<br>Focus groups comprised of 20 participants; 10 mental health care professionals, 5 patients, and 5 family members.	Country: Korea<br>Service: University researchers recruiting participants with depressive experienced, mental health professionals and family members.	The qualitative component of the design process centered the goals of the program on raising awareness of the importance on managing depressive symptoms, promoting mental health, and providing evidence-based interventions.	User-centered design model underpinned intervention design
Barnes E, et al [52]	Focus groups	Focus group 1: 2 health professionals, 2 patients.<br>Focus group 2: 5 professionals, 4 patients. Focus group 3: 3 professionals, 3 patients, and 2 lay persons.	Country: UK<br>Service: university researchers recruiting patients and mental health professionals.	Individual differences, addressing the needs of a wide spectrum of users, relapse prevention, support, and stigma reduction.	No theoretical model used to underpin findings.
Bendelin N, et al [62]	In-depth interviews	Twelve participants, 6 female.<br>Age range: 20 to 62, mean age 36.3 years.	Country: Sweden<br>Service: academic researchers recruited from a randomized control trial that compared two forms of Internet-based CBT for depression.	Working process, motivation, attitudes toward treatment, and consequences of treatment.	Results corresponded with existing theoretical models of change.
Bradley KL, et al [35]	Telephone interviews	Thirteen adolescents (9 girls) took part with ages ranging from 15 to 18 years (M=16.46).	Country: Canada<br>Service: university and health researchers recruited adolescents with mild to moderate depression	Effectiveness, privacy allows control over disclosure and capacity to seek help.	No theoretical model used to underpin findings.
Cheek C, et al [41]	Focus groups	Five focus groups with a total of 16 participants, 12 male and	Country: Australia<br>Service: university	Personalization, engagement, stigma.	No theoretical model used to underpin findings.

Study	Method	Participants	Setting	Summary of findings	Notes
		4 female between the ages of 13 and 18 years old.	research recruiting young people from rural areas who attend community services.		
Clarke JN, Sargent C [58]	Analysis of online message boards	Sixty-six message board discussions including at least 6 interactions in response to a post; 396 posts were analyzed.	Country: Canada<br><br>Service: researchers analyzed message board postings by parents with depressed children used worldwide.	Moods, sociability, behaviors: parents, school, parental ability to cope, causation - social, biological, what can be done about childhood depression.	No theoretical model used to underpin findings.
Danaher BG, et al [61]	Focus groups	Two focus groups with a total of 17 participants. Australia: 8 women with a mean age of 36.0 years. Iowa, US: 9 women with a mean age of 29.4 years.	Country: Australia and the United States<br><br>Service: university research recruiting mothers who were 12 months or less postpartum and had experienced depressive symptoms in that period.	Support for Web forum and partner support features. The use of multicultural video vignettes was endorsed. Encourage skill practice and the use of a personal coach.	No theoretical model used to underpin findings.
Donkin L, Glozier N [53]	Semistructured interviews	Twelve participants aged 45 years and above with at least moderate levels of depression.	Country: Australia<br><br>Service: university researchers recruiting comorbid cancer and depression sufferers.	Barriers and motivations’	No theoretical model used to underpin findings.
Drake G, et al [33]	Focus groups	Two focus groups with 4 and 5 participants in each.	Country: United Kingdom<br><br>Service: university researchers recruited patients from general practitioner surgeries with	Tool was acceptable overall but clarification of the role and target group was required.	No theoretical model used to underpin findings.

Study	Method	Participants	Setting	Summary of findings	Notes
			mild to moderate depression who used the Internet regularly.		
Fleming TM, et al [39]	Focus groups	Five focus groups with a total of 39 young people, 74% males, 49% Maori, 38% Pacific Islanders, and all aged between 13-16 years.	Country: New Zealand<br>Service: university researchers recruited adolescents alienated from mainstream education.	Most were very reluctant to seek help from professionals. High level of interest in computer programs to help with depression. Barriers included lack of help-seeking, access, and potential embarrassment.	No theoretical model used to underpin findings.
Fogel J, Nehmad E [38]	Interviews	Twenty undergraduate students, 10 women and 10 male, Mean age 21.	Country: United States<br>Service: academic researchers recruit undergraduate students.	Using the Internet to obtain information about depression, not using the Internet to obtain information about depression, useful topics to include on an Internet website.	No theoretical model used to underpin findings.
Horgan A, et al [40]	Forum posts	Fifty-three forum posts were analyzed from 17 different users over 3 months.	Country: Ireland<br>Service: researchers created website for students and analyzed forum posts.	Symptoms of depression and loneliness of college life, benefits of website sharing and identifying with others, advice giving, and receiving emotional and informational support.	No theoretical model used to underpin findings.
Iloabachie C, et al [63]	Interviews	Eighty-three adolescents were recruited (56% female). Forty received interviews from their primary care	Country: United States<br>Service: university research recruiting adolescents with subclinical depression and their	Nine themes developed. General sense of improvement in psychosocial function across the intervention. Parent experience/recognition of the	A theoretical model was derived from the quantitative and qualitative analysis.

Study	Method	Participants	Setting	Summary of findings	Notes
		physician and the remainder by the study physician.	parents.	challenges faced by their children in successful and effective completion of the intervention and a desire to be more involved and appreciation of the benefits to their child.	
Kurki M, et al [54]	Focus groups	Two focus groups with a total of 12 participants Age between 34-49 years. Three male and 9 female.	Country: Finland<br>Service: academic researchers recruited from university central hospitals, mental health specialized registered nurses.	Nurses' computer and Internet use in practice, nurses' problems in daily computer and Internet use in practice.	No theoretical model used to underpin findings.
Lillevoll KR, et al [34]	Interviews	Total of 14 patients, 5 were men and ages ranged from 22-61 years.	Country: Norway<br>Service: university research recruiting patients with depression.	Being in treatment: taking action to address one's problem, value of talking to a professional, acquiring relevant knowledge, restructuring new knowledge, actual changes in perceptions and interactions.	No theoretical model used to underpin findings.
Meyer D [59]	Semistructured interviews and focus group	Thirteen student interviewees, 10 of whom went on to create an on-going email focus group.	Country: United Kingdom<br>Service: researcher analyzed interviews and focus group findings to create self-help website for students.	No rich data was presented in findings section of paper.	No theoretical model used to underpin findings.
Nicholas J, et al [56]	Semistructured interviews	Total of 39 participants who did not complete the Web-based program	Country: Australia<br>Service: university	Reasons for attrition: difficulties associated with acute phase of Bipolar	No theoretical model used to underpin findings.

Study	Method	Participants	Setting	Summary of findings	Notes
		were interviewed.	researchers.	disorder, not wanting to think about one's illness, and program factors such as information being too general and not personally tailored were main reasons for nonadherence.	
Pagliari C, et al [51]	Focus groups	Ten focus groups with patients and health professionals were carried out in the United Kingdom, Spain, and Romania. Three focus groups of patients (one in each country) and 7 groups of professionals (2 UK, 2 Spain, and 3 Romania).	Country: United Kingdom, Spain, and Romania<br><br>Service: university research recruiting patients and health professionals working with depression.	Configurability and personalization, trustworthiness, avatar as professional ally, functional role of the avatar, avatar as sensible friend, fear of replacing human interaction, and support.	No theoretical model used to underpin findings.
Parker G, Orman J [36]	Informal comments from survey	Total of 9276 patients.	Country: Australia<br><br>Service: researchers in a research institute recruited patients with depression and their professionals.	Patients: easier to talk to general practitioner. Negative: Length of test and repetition of questions. Professionals: assists with diagnosis, educates client, and helps them understand condition. Negative: patient bias in self-report format, biased toward medical model, induce false sense of security, incompatible with some Web browsers.	No theoretical model used to underpin findings.
Pohjanoska-Mäntylä M, et al [45]	Focus groups	Six focus groups were conducted	Country: Finland	Reasons: obtain a second opinion, verify	No theoretical model used to underpin

Study	Method	Participants	Setting	Summary of findings	Notes
		29 Internet users; 26 female, mean age 47.	Service: academic researcher and health service researcher recruited Internet users with depression to analyses Web-based drug information access.	information within package, prepare for physician appointment and learn about peer experiences. Internet complimented rather than replaced information by professionals.<br><br>Self-reported impacts: increased autonomy, improved knowledge, being reassured, deciding to change dose, discontinue a drug, and to suggest a new drug to a physician.	findings.
Proudfoot J, et al [42]	Focus groups and interviews	Six focus groups with a total of 47 participants. Four were held in urban areas and 2 in rural towns. Of the urban groups, 2 specifically targeted young people aged 18 to 28. Twenty interviews.	Country: Australia<br><br>Service: academic researchers recruited community participants with or without depression, anxiety, or stress.	Current mobile phone behavior, attitudes toward using mobile for mood monitoring, mode of using the program, key functions and features required.	No theoretical model used to underpin findings.
Purves DG, and Dutton J [46]	Interviews	Seven participants, 6 female and an age range of 30-57 years.<br><br>A small homogenous sample in line with interpretive phenomenol ogy-ical analysis methodology .	Country: U.K.<br><br>Service: National Health Service researchers recruiting people with mild depression who had completed Blues Begone without additional human help or support.	The meaningful relationship: being the nature, pattern, and degree of the participant's relationship with the self-help material, and its impact on the participant's psychological process.<br><br>Shape from	No theoretical model used to underpin findings.

Study	Method	Participants	Setting	Summary of findings	Notes
				confusion: the use of structure to bring order, focus, shape, and clarity to their psychological state. Stimulation: stimulation of senses and cognitive activity by content and presentational format of material enhanced engagement.	
Richards D, Timulak L [57]	Qualitative component in questionnaire	Eighty participants: email cognitive behavioral theory had a total of 37 participants, 26 were women and ages ranged from 19-59 years. Computerized cognitive behavior therapy had a total of 43 participants, 25 were woman and ages ranged from 20-50 years.	Country: Ireland<br><br>Service: university research recruiting students with moderate depression.	Helpful events: provision of information, scheduling, monitoring, restructuring, problem solving, and distraction techniques leading to learning new coping skills, behavioral changes, developing awareness, insight, and self-efficacy. Unhelpful events: burden of work, issues with content and form of delivery, time, and pace and technical problems leading to frustration, confusion, irritability, anxiety, disappointment, and being self-critical.	No theoretical model used to underpin findings.
Sobowale K, et al [66]	Focus group	Focus group with 16 bilingual Chinese adolescent participants aged 18-21 years.	Country: China<br><br>Service: university researchers recruiting adolescents with depression, experts,	Behavioral activation, cognitive behavioral therapy, and resilience models were maintained while interpersonal	Behavioral vaccine model was used and significant adaptions to the model were recommended upon

Study	Method	Participants	Setting	Summary of findings	Notes
			school, and parent perspectives to be analyzed using theoretical thematic analysis.	therapy models were excluded from the Chinese adapted intervention. Social worker consultations were identified as the best point of entry for the intervention.	completion of the research.
Stjernswärd S, Östman M [37]	Forum posts and focus group	Forum posts from a 10-week test period were printed out and analyzed with permission from their authors, including posts from dropouts. Three focus groups totaling 5, 4, and 4 participants, respectively.	Country: Sweden<br><br>Service: university researchers recruited participants from a previous study and advertisement s in local newspapers. Previous participants had been recruited through a psychiatric ward, regional newspaper, support organization, and a thread in an online forum.	Diary was found to be a place of discharge, think tank and routines. The forum was a place of discharge, sense of community, exchange, and privacy. The website is seen as a communication tool, communication with the self, and communication with others, advantages, and obstacles.	No theoretical model used to underpin findings.
Stjernswärd S, et al [43]	Focus groups	Focus group 1 totaled 4 participants, 3 women and 1 man.<br><br>Focus group 2 totaled 4 participants, all women.	Country: Sweden<br><br>Service: university researchers recruited participants through a psychiatric ward, regional newspaper, support organization, and a thread in an online forum.	Motivations: to create understanding and rehabilitate oneself and hindrances: lack of time or energy were identified. Design decisions were made on participant's privacy concerns, needs of support and the influence depression has on a relative's life.	No theoretical model used to underpin findings.
Swinton J, et al [47]	Focus groups	Two focus groups. Focus group	Country: United States	Behavioral telehealth is a reasonable	No theoretical model used to underpin

Study	Method	Participants	Setting	Summary of findings	Notes
		1: primary care providers totaling 17 participants, 5 were female and 12 male. Focus group 2: Patients totaling 28 participants, 22 female and 6 male.	Service: academic researchers recruited patients and primary care professionals from 5 rural localities.	solution to the access-to-care problem, the therapeutic relationship is essential to treatment success.	findings.
Takahashi Y, et al [44]	Content analysis	Thirty-seven valid respondents. Median age 37, 43% male.	Country: Japan<br><br>Service: researchers analyzed social networking sites for people with self-reported depressive symptoms.	Advantages: some channels (message, blog, and community) or some functions (invitation/foot print/privacy control functions) ensured advantage conditions like anonymity, easiness, and expectation, creating a place where people could face each other honestly and gain peer support. Disadvantages: solely cyber communication with the social networking sites and intensified dependency by depressed people created additional psychological problems. Potentially trigger downward depressive spiral with social networking sites potentially exacerbating depressive symptoms.	No theoretical model used to underpin findings.
Todd NJ, et al [50]	Focus groups	Twelve service users took part on the first	Country: United Kingdom	Themes identified: gaining an awareness of	No theoretical model used to underpin

Study	Method	Participants	Setting	Summary of findings	Notes
		focus group. Participants were then split in half for focus groups 2 and 3. The average age of participants was 42 years.	Service: university researcher recruited participants with BD from service user groups and on the Internet.	and managing mood swings, not just about Internet, is the only format freely accessible, instant, and interactive, and professional and peer support to overcome low motivation and procrastination.	findings.
Treanor A, et al [64]	Semistructured interviews	Twelve participants with 7 women and a mean age of 33 years for females and 49 years for males.	Country: United Kingdom<br>Service: university, National Health Service, and private sector researchers recruited participants with self-disclosed mood disorder.	Mixed views: some participants were hesitant to use Web-based services due to who could access and view information, whereas others felt very open about sharing information via the Internet. Helped participants recognize patterns in their behavior. Intervention became incorporated into participants' daily routine.	No theoretical model used to underpin findings.
Vayreda A, Antaki C [60]	Conversational analysis	Fourteen forum posts.	Country: Spain<br>Service: researchers analyzed forum posts on a website designed for Spanish speakers with BD.	Giving unsolicited advice although a 'mismatch' is a consequence of the open design of a new user's initial posting. Unsolicited advice might function at the ideological level to induct new users into the group providing support and meaning to bipolar disorder.	No theoretical model used to underpin findings.
Watkins DC, et al [49]	Focus group	One focus group	Country:	Patients had a positive	No theoretical model used to

Study	Method	Participants	Setting	Summary of findings	Notes
		contained 4 men and 4 women.	United States<br><br>Service: university researchers recruited patients with depression from a university-affiliated depression center.	reaction to the idea of an email reminder.	underpin findings.
Whitehill JM, et al [55]	Interviews	Sixty participants, 60% female.	Country: United States<br><br>Service: researchers analyzed structured interviews with college freshman.	Low proportion of depression displayers (30%) wanted a stranger to approach them due to unknown individuals monitoring their Facebook activity.	No theoretical model used to underpin findings.
Wilhelmsen M, et al [48]	Semistructured interviews	Fourteen participants, 9 female with age ranges from 22-61 years.	Country: Norway<br><br>Service: university researchers recruited patients seeking help from their general practitioner for mild to moderate symptoms of depression who had taken part in a randomized control trial of MoodGYM.	Intrinsic motivators were the hope of recovery and a desire to regain control in one's life. An important supporting condition was the ability to freely choose how, when, and where to complete the intervention and satisfied the need for autonomy. A sense of belonging toward family, partner, and friends was an essential motivating factor along with the ability to identify with the intervention.	No theoretical model used to underpin findings.
